# Socioeconomic disparities in survival among follicular lymphoma patients

**DOI:** 10.1038/s41598-026-68993-7

**Published:** 2026-09-15

**Authors:** Susanne von Kroge, Carsten Bokemeyer, Olaf von dem Knesebeck, Fabian Tetzlaff, Alice Nennecke, Annemarie Schultz, Henrik Kusche, Frederik Peters

**Affiliations:** 1 Department of Oncology, Hematology and Bone Marrow Transplantation with Section Pneumology, University Cancer Center Hamburg, University Medical Center Hamburg- Eppendorf, Martinistraße 52, 20251 Hamburg, Germany; 2 Institute of Medical Sociology, University Medical Center Hamburg-Eppendorf, Martinistraße 52, 20251 Hamburg, Germany; 3 Division of Social Determinants of Health, Department of Epidemiology and Health Monitoring, Robert Koch Institute, Nordufer 20, 13353 Berlin, Germany; 4 Hamburg Cancer Registry, Suederstraße 30, 20097 Hamburg, Germany

**Keywords:** Follicular lymphoma, Socioeconomic status, Certified centers, Cancer, Diseases, Health care, Medical research, Oncology, Risk factors

## Abstract

Within a universal healthcare system, the prognostic influence of socioeconomic status (SES) on survival in follicular lymphoma (FL) remains unclear. Thus, we conducted a nationwide, population-based analysis linking cancer registry data with regional SES indicators. Data from patients diagnosed with FL between 2013 and 2023 with mortality follow-up until the end of 2024 were obtained from the German Center for Cancer Registry and linked to the German Index of Socioeconomic Deprivation (GISD) at the district level, which comprises indicators of education, employment, and income. The primary endpoint was relative survival (RS) and secondary endpoint overall survival (OS), comparing patients from low-, middle-, and high-SES districts (categories based on official GISD quintiles). In total, 36 622 FL patients from 400 districts were included. At five years about 80% in female patients and about 77% in male patients were alive. After adjustment for baseline characteristics, females and males in high-SES districts had a 16% and 19% lower excess mortality risk than those in low-SES districts. Further adjustment for degree of urbanity, access to certified centers and a proxy for health-related lifestyle and comorbidities attenuated these differentials considerably. In this first study in a universal healthcare context, SES was an important determinant of survival among FL patients. Proxies for degree of urbanity, access to healthcare and comorbidity at the district-level appeared to account for a substantial part of these differences.

**Supplementary Information:**

The online version contains supplementary material available at https://doi.org/10.1038/s41598-026-68993-7.

## Introduction

With an incidence of 2.4 cases per 100 000 persons per year, follicular lymphoma (FL) is the most common type of indolent non-Hodgkin lymphoma (NHL) in Western Europe and the United States (US), accounting for approximately 20–35% of all NHL cases^1,2^. In Germany, the 10-year prevalence of FL is 13 324 for women and 12 305 for men^3^. Despite a high 5-year relative overall survival (OS) rate of 89%, a considerable number of FL patients die each year from lymphoma-related causes, often after a prolonged disease course^1^. Currently, all NHL risk stratification scores are based solely on biological factors. For FL, these include patient age at diagnosis, number of involved nodal sites, lactate dehydrogenase level, hemoglobin concentration, and disease stage, as integrated by the Follicular Lymphoma International Prognostic Index (FLIPI)^4^. Thus, non-biological factors such as socioeconomic deprivation have not yet been incorporated into lymphoma-specific prognostic models.

The association between low socioeconomic status (SES) and poor health outcomes is internationally well established^5–7^. This also applies to cancer, with patients living in more socioeconomically deprived areas being disadvantaged in terms of incidence, age of disease onset, and survival^8–10^. In a previous analysis of a nationwide German population-based dataset including 49 465 patients diagnosed with diffuse large B-cell lymphoma (DLBCL) between 2004 and 2019, we observed that patients residing in low SES districts had significantly worse 5-year OS compared to those living in middle and high SES districts. This corresponded to a 9% and 15% lower mortality risk in patients living in middle- and high-SES districts, respectively, compared to patients in low SES districts. However, after adjusting for regional premature mortality rates (as a proxy for comorbidities and health-related lifestyle factors), the apparent impact of SES on survival diminished, suggesting that differences in health-related behaviours and comorbidity burden drive disparities in long-term survival among DLBCL patients^11^.

In a universal healthcare system such as Germany’s, with mandatory social health insurance for all its citizens and refugees, legal regulations on health protection, and a high standard of medical care, there are formally no legal differences regarding access to health-related services. Nevertheless, since previous studies investigating the SES impact on FL patients’ survival were mainly conducted in the United Kingdom (UK) and the United States (US)^12–20^, the role of socioeconomic disparities in FL prognosis remains unknown in a universal health care system such as Germany’s. Given our findings in DLBCL, and the American Society of Hematology’s recent Health Equity Compendium highlighting the lack of studies addressing health equity in lymphoma outside the United States noting that only a minority of articles published in Blood, Blood Advances, or Hematology, ASH Educational Program report SES-related data or describe efforts to reduce disparities, we aimed to investigate the prognostic impact of regional SES on long-term survival in FL patients^21^ by conducting a nationwide, registry-based case-control study. To our knowledge, this is the first study to investigate the disease-specific impact of socioeconomic inequalities in FL patients across Germany.

## Materials and methods

### Data sources

This retrospective longitudinal study is based on an evaluated and harmonized pooled cancer registry dataset provided by the Center for Cancer Registry Data at the Robert Koch Institute for the years 2013–2023 covering the whole population of Germany with approximately 84 million inhabitants. The data from the cancer registry is available in different regional resolutions: the 16 federal states at the upper level, corresponding to the Nomenclature des Unités territoriales statistiques (NUTS) hierarchical level 1, and 401 regional districts (territorial status of the year 2020), corresponding to the NUTS-2 level. Furthermore, we extracted aggregated data on the spatial type and premature mortality at the regional level (NUTS-2) from the ongoing spatial observation of the Federal Institute for Research on Building, Urban Affairs, and Spatial Development^22^. Data on regional deprivation at the NUTS-2 level per calendar year are based on the German Index of Socioeconomic Deprivation (GISD) of the Robert Koch Institute^23,24^. Information on the location of certified centers per ZIP code was extracted from the database of the German Cancer Society (Deutsche Krebsgesellschaft - DKG), online available at www.oncomap.de (data accessed at 2026-07-10)^25^. The term certified centers includes certification by the DKG, European Cancer Center, or by ÄKzert^®^ - Zertifizierungsstelle der Ärztekammer Westfalen–Lippe. The extracted list comprises centers certified for haematological neoplasms and reflects the certification status at the time of data access. As no information on the calendar year in which certification for each center began or ended was available, this list represents the certification status in 2026 only. Finally, data on age- and sex-specific one-year death probabilities for the general population based on life tables at the level of Germany were extracted from the Human Mortality Database of the Max Planck Institute for Demographic Research, University of California, Berkeley, and French Institute for Demographic Studies^26^.

### Patients

We included all patients with a primary FL diagnosis coded as “C82” according to the International Statistical Classification of Diseases, German Modification (ICD-10-GM) between January 1st 2013 and December 31st, 2023, with vital status follow-up until December 31st, 2024. Exclusion criteria were age at diagnosis below 20 and above 99 and invalid district ID. Patients with a follow-up duration of less than three months were excluded to remove cases that entered the registry data only from information on death certificates, to avoid immortal time bias as reports up to three months of the initial report to the cancer registry were employed to specify the diagnosis and to focus on the impact of SES on long-term survival rather than on death due to acute conditions.

### Study variables

At the patient level, study variables were place of residence (numbered from 1 to 16 according to federal state of the district), calendar year of diagnosis in years (2013–2023), age at diagnosis in years, sex (female, male), tumor grading according to the classification of the World Health Organization (ICD-O-3 histology code 9695: grade 1, 9691: grade 2, 9698: grade 3)^27^, and a history of other primary tumors (none, one or more). SES was measured using the GISD, which is an area-level deprivation index summarizing aggregated information on employees with a university degree, employees without education, school leavers without qualification, employment rate, unemployment rate, gross earnings, income tax, household income, and debtor quota^23,24^. The GISD score was linked to each case in the cancer registry dataset by district ID and calendar year. Based on the quintiles of the distribution of the GISD, SES was grouped into low (5th quintile), middle (2nd to 4th quintile), and high (1st quintile). This classification aims to clearly distinguish between the two extremes of the socioeconomic spectrum whilst ensuring a statistically stable reference group in the middle. Furthermore, this categorization of the GISD value is consistent with the RKI’s official recommendation on the use of the GISD^23,24^. Earlier work confirmed the suitability of the index to study socioeconomic variations in cancer mortality and in lymphoma in Germany^9,11,28^.

### Outcomes

The primary outcome of the study was relative survival (RS), defined as transformed survival time from the date of diagnosis until the date of death. RS is an established proxy for disease-specific survival, enabling the indirect estimation of the disease burden by carefully adjusting for age-, sex-, and calendar year-specific population mortality rates. This approach effectively removes the influence of background mortality, thereby isolating the excess hazard attributable to the disease when explicit cause-of-death data are unavailable^29^. As a secondary outcome, OS was used, defined as death due to any cause during the study time, thus also including deaths potentially unrelated to FL. Patients were followed until the date of death, the end of the study period, or 1 827 days, whichever occurred first.

### District-level covariates

Districts were categorized using an official spatial typology of the Federal Institute for Research on Building, Urban Affairs, and Spatial Development reflecting their settlement structure here denoted as spatial type and grouped into “rural sparsely”,“rural densely”,“urban” and “larger city” (Siedlungsstruktureller Raumtyp des Bundesinstituts für Bau-, Stadt- und Raumforschung (BBSR)^22^. While this variable was used as a proxy for general access to public services due to a higher degree of urbanity, we further included a variable on the presence of at least one cancer center certified for haematological neoplasms in the district of the residence of a patient (assignment for a district over the whole study period irrespective of the date of first certification because no information on the calendar year in which certification for each center began or ended was available). Premature mortality (defined by the number of deaths per 1 000 residents before age 70 per district) was used as a proxy for the regional comorbidity burden, based on the hypothesis that the general comorbidity burden also affects patients with lymphoma and that this relationship varies according to SES^18^.

### Ethics approval and consent to participate

For this analysis, ethics committee review and informed consent are not required according to federal law. According to Sect.  6, Paragraph 2 of the HmbKrebsRG, the Hamburg Cancer Registry may participate in scientific studies on cancer if no individual can be identified. Moreover, the Hamburg Cancer Registry has permission to use data from other state cancer registries for this purpose. We confirm that all analyses were performed in accordance with relevant guidelines and regulations.

### Statistical analysis

All analyses were performed using R, version 4.3.1, using the packages relsurv, survival, survminer, forester, data.table, and tableone. Categorical variables were summarized as counts and proportions, and continuous variables as medians with interquartile ranges. Unadjusted OS by SES group was estimated using Kaplan-Meier methods. Transformed survival time, expressing excess mortality in relation the general population, was computed using the *relsurv* package and lifetables for the German population from the Human Mortality Database. Continuous variables age and premature mortality were scaled before multivariate analysis. Associations between SES and RS (primary outcome) and OS (secondary outcome) were evaluated in multivariable Cox proportional hazards models adjusted for sex, SES group, age at diagnosis, WHO grading, and history of other tumors. Models incorporated spatial fixed effects for Germany’s 16 federal states to account for time-invariant regional differences, such as quality of cancer registration, and calendar year fixed effects to adjust for temporal confounding. Cluster-robust standard errors at the district level were applied to account for within-district correlation.

In subsequent models, spatial type, presence of certified cancer centers and district-level comorbidity burden were included to assess the extent to which they attenuated the SES-survival association. Results are presented as hazard ratios (HRs) with 95% confidence intervals (CIs). The proportional hazards assumption was evaluated visually using scaled Schoenfeld residuals. Analyses were conducted separately for female and male patients to explore potential sex-specific effects.

For reporting our results, we adhered to the Strengthening the Reporting of Observational Studies in Epidemiology (STROBE) statement^30^. We did not apply adjustments for multiple testing nor report p-values, as all results represent associations observed at the population level rather than estimates derived from sampling variability^31^. The maps were generated using the R package sf for spatial data manipulation and the tmap package for thematic mapping and visualization. The spatial data, sourced from the VG2500 dataset of the Federal Agency for Cartography and Geodesy (BKG), was transformed to align with the EPSG:4314 coordinate reference system, ensuring consistency with the BKG’s reference system. Additionally, the data used adheres to the “Data licence Germany – attribution – Version 2.0.

## Results

**Fig. 1:**
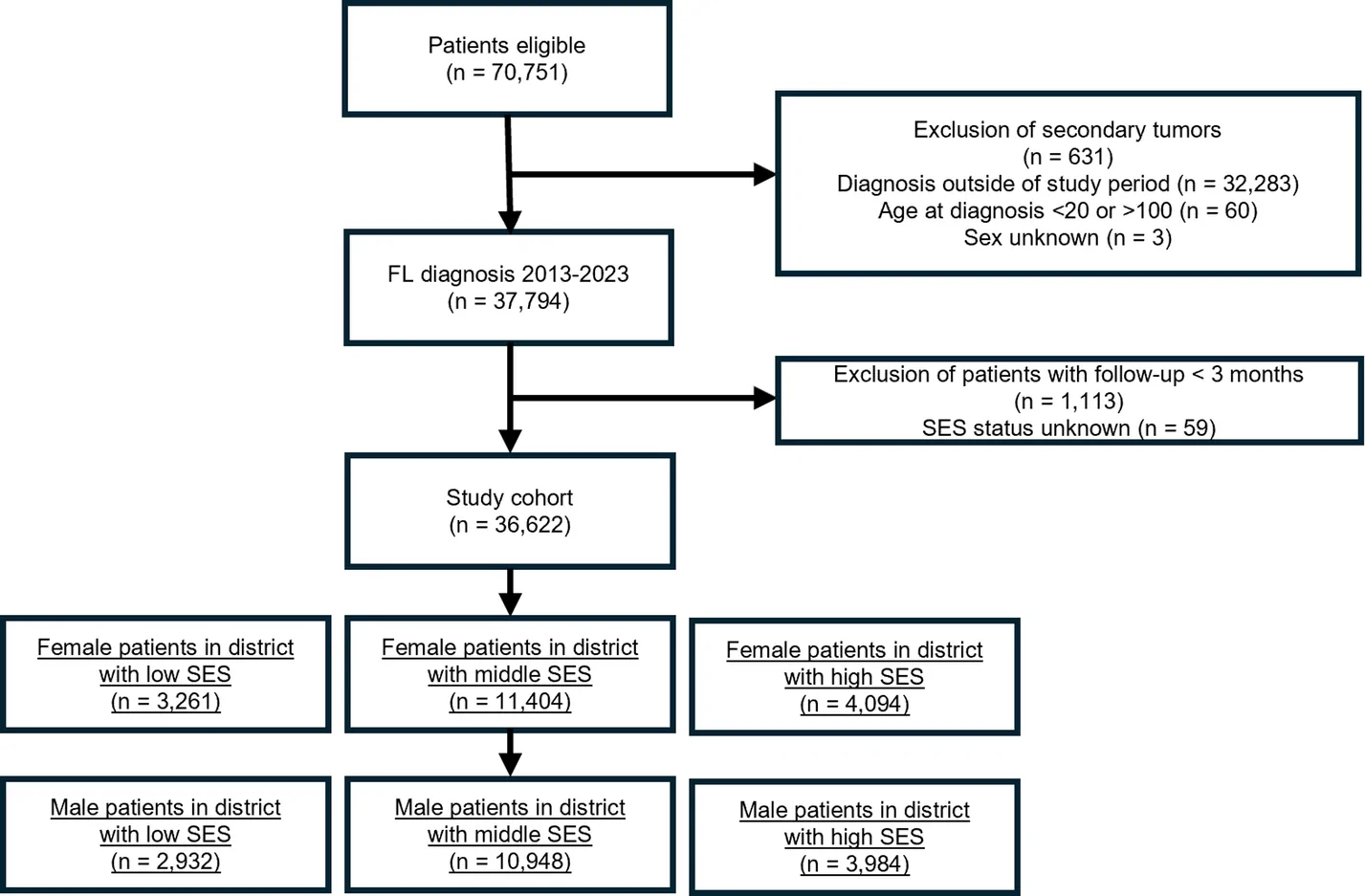
Flow chart. FL: Follicular lymphoma, GISD: German Index of Socieoeoconomic Deprivation, SES: Socioeconomic status.

**Fig. 2:**
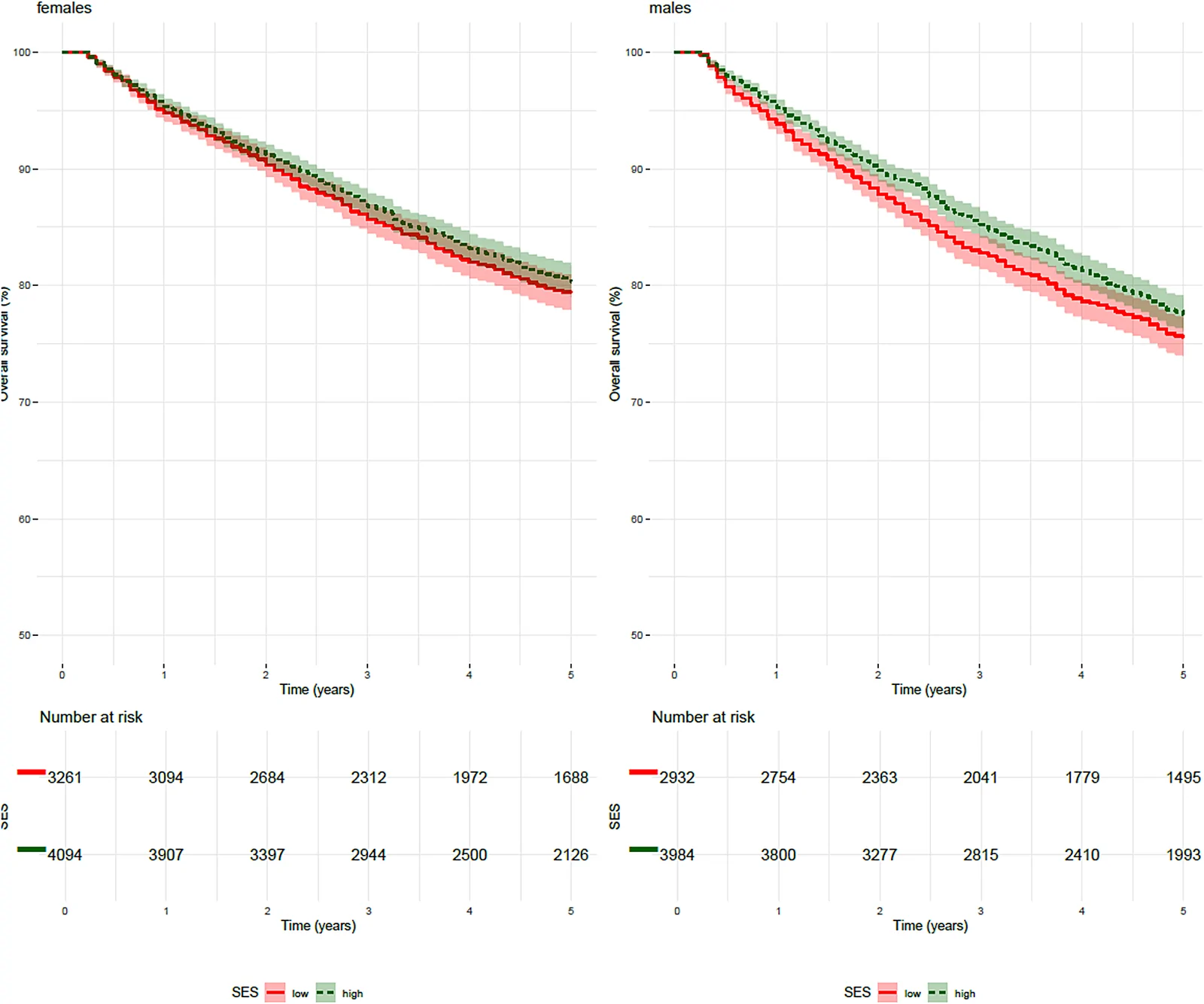
Unadjusted five-year overall survival with 95% confidence intervals in female and male patients with follicular lymphoma; stratified by socioeconomic status (SES). Note: For clarity, the figure is limited to a comparison of the ‘low’ and ‘high’ categories. The complete figures, including the ‘middle’ category, can be found in Web Supplement Figure S5.

In total, 36 622 patients with a primary diagnosis of FL were included in the final analysis (median age 67, 51.2% female, median follow-up 5.0 years, Fig. 1). Thereby, 3 261 female patients and 2 932 male patients lived in districts with low SES, and 4 094 female patients and 3 984 male patients lived in districts with high SES (Table 1). At the time of primary diagnosis, females were, on average, about two years older than males (68 vs. 66 years). Around 11% of patients had at least one other tumor prior to the FL diagnosis with slightly higher rates in males. Patients in high SES districts exhibited a more favorable WHO grading, lived more often in more urban districts, districts with presence of a certified center in a central region, and a lower premature mortality (about 2.3 per 1 000 persons vs. about 3.7 per 1 000 persons) than those in low SES districts. At five years of follow-up, slightly less than 80% of patients survived, which was lower in males and patients in low SES districts (Fig. 2).

**Table 1:** Baseline characteristics follicular lymphoma patients by sex and socioeconomic status.

Females	Socioeconomic status		
	Low (*N* = 3,261)	Middle (*N* = 11,404)	High (*N* = 4,094)
Age in years, median (Q1, Q3)	68 (58, 75)	68 (58, 76)	68 (58, 76)
At least one prior tumor, n (%)	297 (9.1)	1127 (9.9)	395 (9.6)
WHO Grade 1, n (%)	731 (22.4)	2396 (21.0)	893 (21.8)
WHO Grade 2, n (%)	1237 (37.9)	4475 (39.2)	1768 (43.2)
WHO Grade 3*, n (%)	552 (16.9)	1925 (16.9)	613 (15.0)
WHO Grade unknown, n (%)	741 (22.7)	2608 (22.9)	820 (20.0)
Median follow-up in years (Q1, Q3)	5 (2.63, 5)	5 (2.63, 5)	5 (2.71, 5)
Spatial type rural sparsely, n (%)	1129 (34.6)	1814 (15.9)	194 (4.7)
Spatial type rural densely, n (%)	630 (19.3)	2181 (19.1)	445 (10.9)
Spatial type rural urban, n (%)	491 (15.1)	4949 (43.4)	1894 (46.3)
Spatial type rural larger city, n (%)	1011 (31.0)	2460 (21.6)	1561 (38.1)
At least one certified hospital center for haematological neoplasms in district, n (%)	1080 (33.1)	4478 (39.3)	1964 (48.0)
District level deaths before age 70, per 100.000 persons, median (Q1, Q3)	3.78 (3.46, 4.26)	2.90 (2.65, 3.28)	2.30 (2.11, 2.51)

Males	Low (*N* = 2,932)	Middle (*N* = 10,947)	High (*N* = 3,984)
Age in years, median	66 (56, 74)	66 (56, 75)	66 (55, 75)
At least one prior tumor, n (%)	330 (11.3)	1274 (11.6)	474 (11.9)
WHO Grade 1, n (%)	634 (21.6)	2317 (21.2)	881 (22.1)
WHO Grade 2, n (%)	1091 (37.2)	4278 (39.1)	1703 (42.7)
WHO Grade 3*, n (%)	496 (16.9)	1809 (16.5)	586 (14.7)
WHO Grade unknown, n (%)	711 (24.2)	2543 (23.2)	814 (20.4)
Median follow-up in years	5 (2.55, 5)	4.88 (2.50, 5)	5 (2.63, 5)
Spatial type rural sparsely, n (%)	919 (31.3)	1655 (15.1)	204 (5.1)
Spatial type rural densely, n (%)	560 (19.1)	2156 (19.7)	417 (10.5)
Spatial type rural urban, n (%)	511 (17.4)	4882 (44.6)	1859 (46.7)
Spatial type rural larger city, n (%)	942 (32.1)	2254 (20.6)	1504 (37.8)
Al least one certified hospital center for haematological neoplasms in district, n (%)	949 (32.4)	4123 (37.7)	1902 (47.7)
District level deaths before age 70, per 100.000 persons, median (Q1, Q3)	3.72 (3.42, 4.16)	2.90 (2.65, 3.27)	2.31 (2.11, 2.51)

Abbreviations: Q1: First quartile, Q3: Third quartile, WHO: World Health Organization.

*No data are available on the subdivision into grades 3A and 3B.

**Fig. 3:**
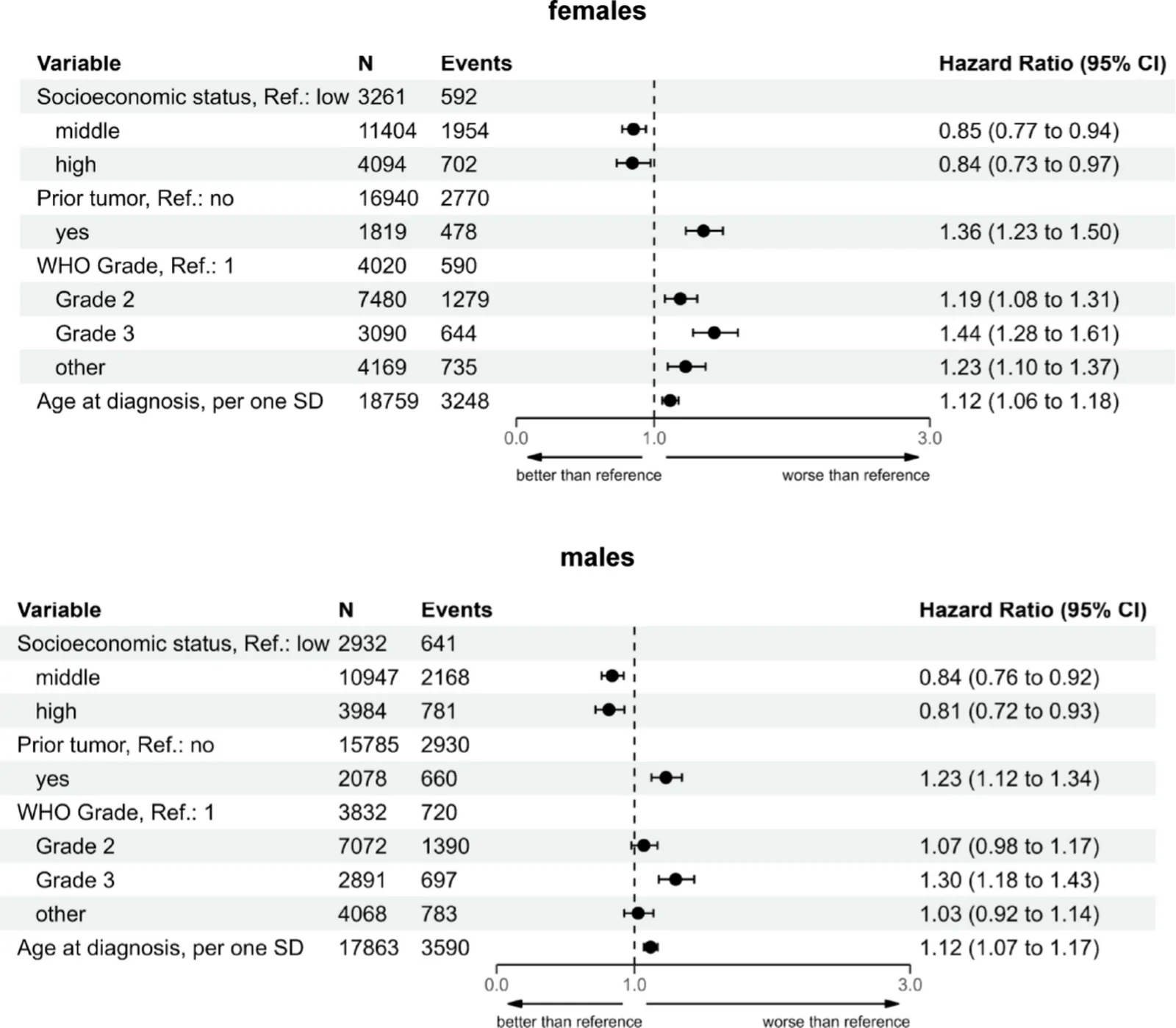
Five-year relative survival with 95% confidence intervals in female and male patients with follicular lymphoma adjusted for patient characteristics and tumor factors only. WHO: World Health Organization, SD: Standard Deviation.

After adjustment for baseline characteristics, excess mortality risk in female patients in middle SES and high SES districts was each about 15% and 16% lower than that in low SES districts (HR 0.85 (95% CI: 0.77–0.94) and 0.84 (95% CI: 0.73–0.97), top panel, Fig. 3). In males, the relative difference was even more pronounced at 16% for patients in middle SES districts and 19% for patients in high SES districts (HR 0.84 (95% CI: 0.76–0.92) and 0.81 (95% CI: 0.72–0.93), bottom panel, Fig. 3). In all models, higher age, WHO grading 3, and a history of other prior tumors were associated with a higher excess mortality risk.

**Fig. 4:**
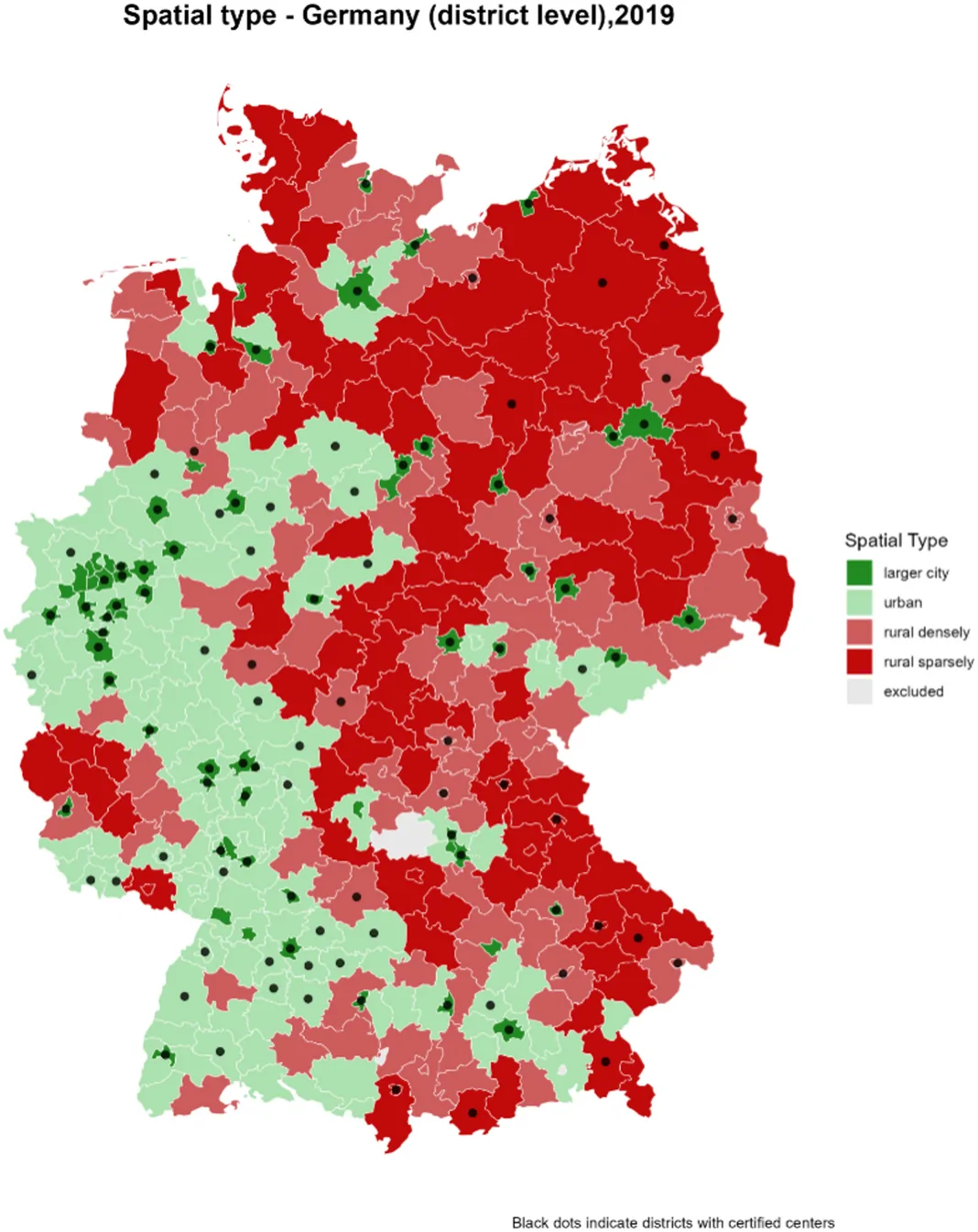
Regional distribution of districts by spatial type in 2019; the black dots represent districts with at least one certified hematological center (certification by the DKG, European Cancer Center, or by ÄKzert^®^ - Zertifizierungsstelle der Ärztekammer Westfalen–Lippe). The maps were generated using the R package sf for spatial data manipulation and the tmap package for thematic mapping and visualization R, Version 4.3.1.). The spatial data, sourced from the VG2500 dataset of the Federal Agency for Cartography and Geodesy (BKG), was transformed to align with the EPSG:4314 coordinate reference system, ensuring consistency with the BKG’s reference system. Additionally, the data used adheres to the “Data licence Germany – attribution – Version 2.0. (https://gdz.bkg.bund.de/index.php/default/verwaltungsgebiete-1-2-500-000-stand-31-12-vg2500-12-31.html).

**Fig. 5:**
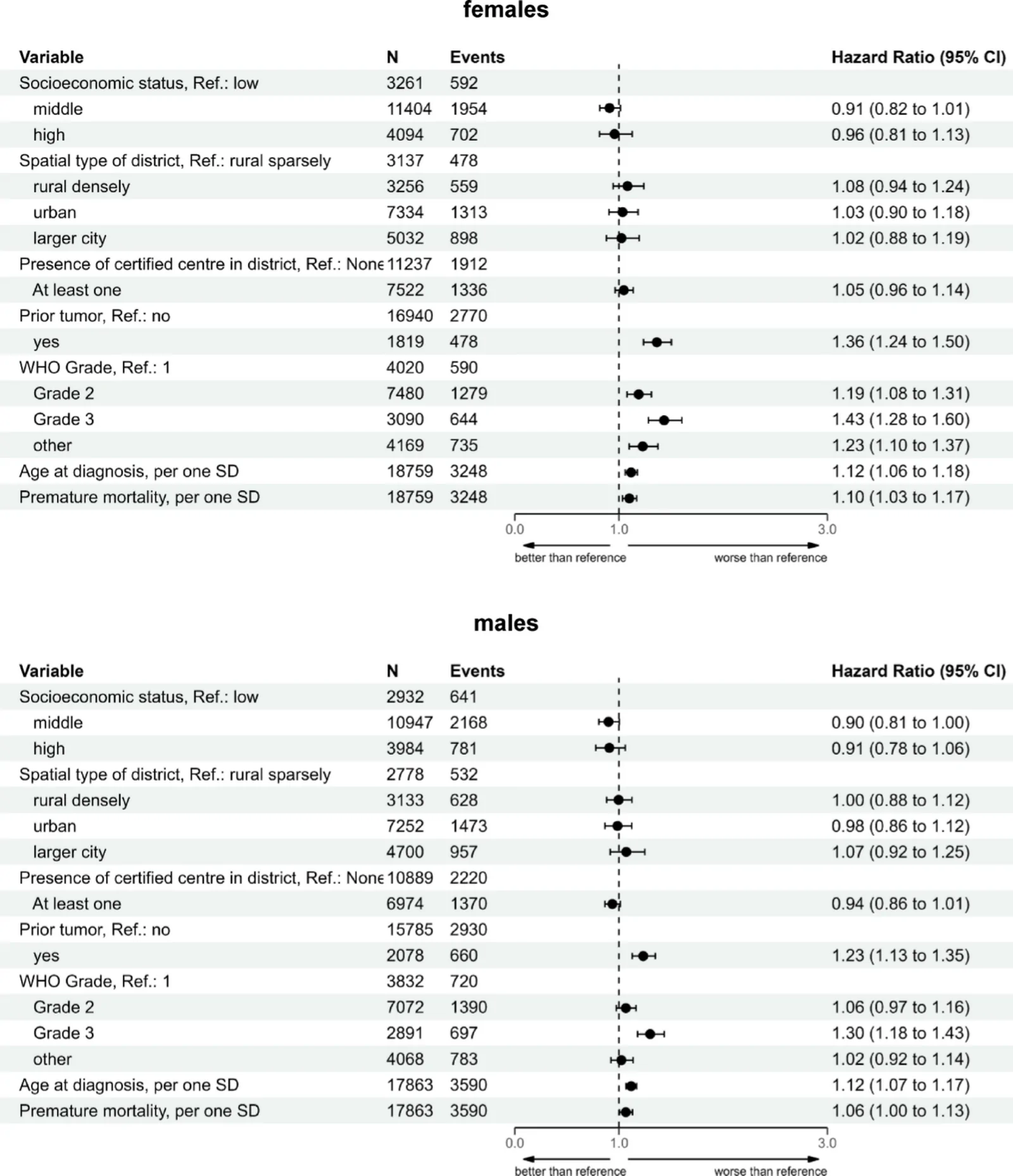
Five-year relative survival with 95% confidence intervals in female and male patients with follicular lymphoma, adjusted for patient characteristics, tumor factors and urbanity and proxies of access to high-quality care and comorbidity burden. WHO: World Health Organization, SD: Standard Deviation.

After further adjustment for the influence of urbanity (Fig. 4), presence of high-quality care and comorbidity burden, the difference between female patients living in middle SES and high SES as compared to low SES districts diminished to 9% and 4% (HR 0.91 (95% CI: 0.82–1.01) and 0.96 (95% CI: 0.81–1.13), top panel, Fig. 5). This was comparable in males, where differences diminished slightly to 10% and 9% (HR 0.90 (95% CI: 0.81-1.00) and 0.91 (95% CI: 0.78–1.06), bottom panel, Fig. 5). In male and female patients a higher degree of district-level premature mortality by one standard deviation was associated with a slightly higher excess mortality risk. Thereby, the association appeared to be a bit larger in female patients (HR 1.10, 95% CI: 1.03–1.17) than in male patients (HR 1.06, 95% CI: 1.00-1.13). Interestingly, there was no sizable association with excess mortality risk and the degree of urbanity and presence of a certified center.

All main results were confirmed by analyses using overall survival as the endpoint (refer to web supplement Figures S3 and S4). Age had a stronger association in these analyses than in the primary endpoint of excess mortality.

## Discussion

By reporting the results of a large nationwide study analyzing the impact of regional SES on FL patients’ survival in the setting of a universal healthcare system, including more than 36 000 FL patients, we present the largest European study population reported to date. After adjusting for baseline characteristics, we observed considerable SES-related survival differences: patients residing in low SES districts had a lower survival probability following FL diagnosis compared with those in districts with middle or high SES. These differences were slightly more expressed in men than in women. After adjusting for the influence of urbanity, access to high-quality care and premature mortality (proxies for proximity of high-quality care and comorbidity burden), SES differences did diminished considerably.

By observing a higher excess mortality risk in FL patients living in lower SES districts compared to those living in middle or higher SES districts, our results align with results previously reported from the UK and US^12,14,15,17,19^. In the UK, Smith et al. reported that FL patients living in the most deprived districts of England had 1.45-fold higher excess mortality compared to those living in the least deprived districts^12^. Similarly, in the State of California, Keegan and colleagues observed a 1.44-fold higher excess mortality in FL patients living in the lowest neighborhood SES group compared to the higher SES group^15^. To the best of our knowledge, no other SES-related survival data on FL patients are currently available from countries other than the UK and the US. The variation in excess mortality observed in our study (up to 19% in males) compared to the approximately 45% previously reported may, in part, be explained by differences in the periods analyzed, as survival in FL patients has generally improved over recent decades^15,19^. Although Keegan et al. and Smith and colleagues observed improvements in OS over time, their study periods (1988–2005 and 2005–2013, respectively) did not include more recent therapeutic innovations, such as rituximab maintenance treatment for FL patients, the combination of obinutuzumab and chemotherapy, followed by subsequent obinutuzumab maintenance for first-line therapy of high tumor burden FL or the combination of rituximab and involved-field radiotherapy in early-stage FL^32–34^. Moreover, the absence of an internationally standardized definition of (regional) SES and the utilization of different, not harmonized deprivation concepts complicate cross-study comparisons. Additionally, there are significant differences in health policy and health insurance systems between the UK, the US, and Germany, as these countries have public, private, and social health systems, respectively^35,36^. It is noteworthy, however, that SES-related differences in survival among FL patients were observed in all these health insurance systems, with the potential impact on survival rates being lowest in Germany, followed by the US and the UK. Nevertheless, based on the reasons mentioned above, a direct comparison of the study results is considerably limited.

Assuming that an important mechanism between regional socioeconomic deprivation and long-term survival in FL patients is the structural composition of the regional unit, our results are consistent with findings from the US. By analyzing data from 43 393 FL patients in the US National Cancer Data Base, Ritter et al. reported that individuals living in urban and rural areas (hereafter referred to as non-metropolitan areas) were less likely to have private insurance and generally had a lower SES. These patients showed worse OS and were more often treated in community-based settings. In addition, FL patients living in non-metropolitan areas differed from their metropolitan counterparts by being older, more frequently male, white, with lower income, higher comorbidity burden, and delays in treatment initiation^13^. However, unlike in the US, a lack of access to healthcare coverage, which is known to negatively impact FL patients’ survival in the US, is prevented in Germany through compulsory health insurance mandated by law^37^. Perhaps for that reason, the degree of district-level urbanization itself did not indicate unwarranted variation in outcomes in addition to the other confounders in our data. For the presence of a certified center, the models likewise did not establish a clear association with excess mortality. At first sight, this appears to contrast with earlier findings of a protective effect of treatment in certified centers for various cancers in Germany^38^. Those findings, however, compared patients treated within versus outside certified centers and were therefore susceptible to channeling bias^39^. Our variable differs conceptually: it is an area-level (ecological) measure of the geographic presence of a certified center in the district of residence. While this ecological definition avoids channeling bias at the individual level, it does not eliminate referral, selection, or residual confounding related to regional healthcare structures, because patients may be treated outside their district, referred to a certified center elsewhere, or not use a locally available center. Also, the most recent data on regional certification status was used for all patients throughout the study time, introducing a certain degree of misclassification if patients were treated before a later certification. Thus, the null estimate cannot exclude attenuation caused by exposure misclassification.

Generally, the negative impact of a higher comorbidity burden on cancer survival is well known^40^. However, it remains unclear whether undertreatment, possibly due to higher treatment-related toxicity risks or poor therapy adherence among patients with comorbidities, contributes to these disparities^40^. This also applies in the context of FL. The prognostic impact of comorbidity burden on survival has been previously reported for FL patients^12,37^. However, mediating mechanisms as well as which specific comorbidities, or combinations thereof, have the most significant impact on FL survival remain unknown. Moreover, sex-specific disparities have been observed regarding FL patients’ survival, with females generally having better OS compared to males^15^. However, Nabhan et al. reported that these differences diminished over time (1992–2000 vs. 2001–2009)^19^. Whether a possible sex-dependent efficacy of rituximab contributes to these findings remains unclear^19^. Even though previous analyses have focused on key factors, such as the influence of race, regional SES, sex, or comorbidity burden, our study aimed to investigate the mechanisms through which regional SES may influence survival differences in FL patients in recent years. Our findings demonstrate that SES differentials in survival seem to be influenced by district-level factors in both female and male patients to a comparable extent. Whether the small remaining differences actually point to a higher degree of vulnerability to socioeconomic deprivation in males might be subject to further studies, also including more detailed information on cancer biology, treatment, lifestyle, and comorbidities. This might help to disentangle the impact of the different aspects more precisely than in our data where ecological information on urbanity (Fig. 4), SES (Web Supplement Figure S1), quality of care and comorbidities (Web Supplement Figure S2) overlapped to a large degree.

This epidemiological study is based on cancer registry data, which include general information on patient demographics and tumor characteristics, but lack more detailed prognostic variables such as Ann Arbor stage or FLIPI, as well as individual-level data on treatment, comorbidities, lifestyle factors, as well as determinants of socioeconomic status at the individual level (e.g. educational attainment, income, or occupational attainment). Moreover, although histological gradings are equally distributed across all SES groups, there is no data available on the subdivision of grade 3 into 3 A and 3B. To address potential confounding, we included fixed effects for calendar year and federate state into the models, to indirectly adjust for unobserved patient characteristics that change over time or differ between places or are related to the fact that not all local registries provided full coverage of FL patients over the whole study time or other aspects of cancer registration that changed over time. As previously reported in our analysis of DLBCL patients^11^, this study is limited by the fact that the central conclusions are based on all-cause mortality and that causes of death were not available at sufficient quality in the data. However, we therefore modeled relative survival as a common proxy for disease-specific survival so that more detailed information on e.g. cause of death was not necessary^11^. We are confident that by incorporating various data sources (e.g., insurance billing data) and accessing more detailed federal cancer registry data, we will soon be able to provide a more detailed explanation of the relationship between survival, comorbidity burden, and structural district-level characteristics in FL patients.

Despite the absence of legal barriers to healthcare access in Germany, our results indicate that socioeconomic deprivation has a significant negative impact on the long-term prognosis of FL patients. This association may be attenuated by structural district-level characteristics, i.e. the burden of comorbidities, and health-related lifestyle behaviours. If further studies using individual-level patient data and the analysis of patient pathways confirm our hypothesis that structural characteristics drive the increased mortality risk among FL patients living in low SES districts, targeted public health interventions such as second opinion services, telemedicine, and outreach partnerships will be needed to reduce socioeconomic disparities in FL patient outcomes.

## Supplementary Information

Below is the link to the electronic supplementary material.

Supplementary Material 1

Supplementary Material 2

Supplementary Material 3

## Data Availability

The data included in the study were analyzed in the protected environment of the Hamburg Cancer Registry. Due to data protection and security regulations, the sensitive patient data cannot be made publicly available. Generally, however, data for scientific projects can be requested from the Hamburg Cancer Registry. All analysis scripts can be made available on request from the corresponding author.
