# Supplementary material for: Socioeconomic disparities in survival among follicular lymphoma patients
Source: Sci Rep. 2026 Sep 15;16:28763. doi: 10.1038/s41598-026-68993-7 (PMC13578211; doi:10.1038/s41598-026-68993-7)
Supplement: Supplementary file 2 [file 41598_2026_68993_MOESM2_ESM.docx]

**Web supplement**

Figure S1. Regional distribution of districts by socioeconomic status in 2019; the black dots represent districts with at least one certified hematological center (certification by the DKG, European Cancer Center, or by ÄKzert® - Zertifizierungsstelle der Ärztekammer Westfalen–Lippe).

Figure S2. Regional distribution of districts by premature mortality in 2019; the black dots represent districts with at least one certified hematological center (certification by the DKG, European Cancer Center, or by ÄKzert® - Zertifizierungsstelle der Ärztekammer Westfalen–Lippe).

Figure S3. Five-year overall survival with 95% confidence intervals in female and male patients with follicular lymphoma adjusted for patient characteristics and tumor factors only. WHO: World Health Organization, SD: Standard Deviation.

**females**

**males**

Figure S4. Five-year overall survival with 95% confidence intervals in female and male patients with follicular lymphoma, adjusted for patient characteristics, tumor factors and urbanity and proxies of access to high-quality care and comorbidity burden. WHO: World Health Organization, SD: Standard Deviation.

**females**

**males**

Figure S5. Unadjusted five-year overall survival with 95% confidence intervals in female and male patients with follicular lymphoma; stratified by socioeconomic status (SES).
