# Supplementary material for: Socioeconomic disparities in survival among follicular lymphoma patients
Source: Sci Rep. 2026 Sep 15;16:28763. doi: 10.1038/s41598-026-68993-7 (PMC13578211; doi:10.1038/s41598-026-68993-7)
Supplement: Supplementary file 3 [file 41598_2026_68993_MOESM3_ESM.docx]

**Web supplement**

Figure S1. Regional distribution of districts by socioeconomic status in 2019; the black dots represent districts with at least one certified hematological center (certification by the DKG, European Cancer Center, or by ÄKzert® - Zertifizierungsstelle der Ärztekammer Westfalen–Lippe). The maps were generated using the R package sf for spatial data manipulation and the tmap package for thematic mapping and visualization R, Version 4.3.1.). The spatial data, sourced from the VG2500 dataset of the Federal Agency for Cartography and Geodesy (BKG), was transformed to align with the EPSG:4314 coordinate reference system, ensuring consistency with the BKG’s reference system. Additionally, the data used adheres to the “Data licence Germany – attribution – Version 2.0. (<https://gdz.bkg.bund.de/index.php/default/verwaltungsgebiete-1-2-500-000-stand-31-12-vg2500-12-31.html>)

Figure S2. Regional distribution of districts by premature mortality in 2019; the black dots represent districts with at least one certified hematological center (certification by the DKG, European Cancer Center, or by ÄKzert® - Zertifizierungsstelle der Ärztekammer Westfalen–Lippe). The maps were generated using the R package sf for spatial data manipulation and the tmap package for thematic mapping and visualization R, Version 4.3.1.). The spatial data, sourced from the VG2500 dataset of the Federal Agency for Cartography and Geodesy (BKG), was transformed to align with the EPSG:4314 coordinate reference system, ensuring consistency with the BKG’s reference system. Additionally, the data used adheres to the “Data licence Germany – attribution – Version 2.0. (<https://gdz.bkg.bund.de/index.php/default/verwaltungsgebiete-1-2-500-000-stand-31-12-vg2500-12-31.html>)

Figure S3. Five-year overall survival with 95% confidence intervals in female and male patients with follicular lymphoma adjusted for patient characteristics and tumor factors only. WHO: World Health Organization, SD: Standard Deviation.

**females**

**males**

Figure S4. Five-year overall survival with 95% confidence intervals in female and male patients with follicular lymphoma, adjusted for patient characteristics, tumor factors and urbanity and proxies of access to high-quality care and comorbidity burden. WHO: World Health Organization, SD: Standard Deviation.

**females**

**males**

Figure S5. Unadjusted five-year overall survival with 95% confidence intervals in female and male patients with follicular lymphoma; stratified by socioeconomic status (SES).
